# Parkinson’s disease in primary health care and nursing care: a scoping review

**DOI:** 10.1590/1980-220X-REEUSP-2021-0367

**Published:** 2022-03-11

**Authors:** Simony Fabíola Lopes Nunes, Angela Maria Alvarez, Rafaela Vivian Valcarenghi

**Affiliations:** 1 Universidade Federal do Maranhão, Departamento de Enfermagem, Imperatriz, MA, Brazil.; 2 Universidade Federal de Santa Catarina, Departamento de Enfermagem, Florianópolis, SC, Brazil.; 3 Prefeitura Municipal de São José, São José, SC, Brazil.

**Keywords:** Nursing, Nursing Care, Noncommunicable Diseases, Parkinson Disease, Primary Health Care, Review, Enfermería, Atención de Enfermería, Enfermedades no Transmisibles, Enfermedad de Parkinson, Atención Primaria de Salud, Revisión, Enfermagem, Cuidados de Enfermagem, Doenças Não Transmissíveis, Doença de Parkinson, Atenção Primária à Saúde, Revisão

## Abstract

**Objective:**

to map and analyze the scientific literature on nursing care aimed at people with Parkinson’s disease in Primary Health Care.

**Method:**

this is a scoping review using the Joanna Briggs Institute methodology, carried out between July and October 2020, and updated in November 2021 in six databases from nursing care and Parkinson’s disease descriptors, and their respective acronyms and synonyms in English, Portuguese and Spanish.

**Results:**

a total of 44 publications were included in this review, which identified as nursing care in Primary Care: assessment of motor and non-motor functions; management of activities of daily living and instrumental activities of daily living; disease self-management education for people with Parkinson’s and their care partners; supervised group approach; and personal factor management.

**Conclusion:**

Nursing care for people with Parkinson’s at the primary level essentially involves actions that include providing focused care at an individual and group level, encompassing clinical assessment, patient education, patient involvement in the social context of care, and developing positive relationships with family members and caregivers.

## RESUMO

**Objetivo:**

mapear e analisar a literatura científica acerca dos cuidados de enfermagem direcionados às pessoas com doença de Parkinson na Atenção Primária à Saúde.

**Método:**

revisão de escopo utilizando a metodologia do *Joanna Briggs Institute*, realizada entre julho e outubro de 2020, e atualizada em novembro de 2021 em seis bases de dados, a partir dos descritores cuidados de enfermagem e doença de Parkinson, e seus respectivos acrônimos e sinônimos em inglês, português e espanhol.

**Resultados:**

foram incluídas 44 publicações nesta revisão, que identificaram como cuidados de enfermagem na Atenção Primária: avaliação das funções motoras e funções não motoras; gerenciamento das Atividades de Vida Diária e das Atividades Instrumentais de Vida Diária; educação para autogestão da doença para pessoas com Parkinson e seus parceiros de cuidado; abordagem supervisionada em grupo; e gerenciamento dos fatores pessoais.

**Conclusão:**

os cuidados de enfermagem à pessoa com Parkinson no âmbito primário envolvem, essencialmente, ações que incluem prestar cuidados centrados em nível individual e em grupo, abrangendo avaliação clínica, educação do paciente, envolvimento deste no contexto social do cuidado e desenvolvimento de relacionamentos positivos com os familiares e cuidadores.

## RESUMEN

**Objetivo:**

mapear y analizar la literatura científica sobre el cuidado de enfermería dirigido a personas con enfermedad de Parkinson en la Atención Primaria de Salud.

**Método:**

una revisión del alcance utilizando la metodología del *Joanna Briggs Institute*, realizada entre julio y octubre de 2020, y actualizada en noviembre de 2021 en seis bases de datos, de los descriptores cuidado de enfermería y enfermedad de Parkinson, y sus respectivas siglas y sinónimos en inglés, portugués y español.

**Resultados:**

en esta revisión se incluyeron 44 publicaciones, que identificaron como cuidados de enfermería en Atención Primaria: evaluación de las funciones motoras y no motoras; gestión de Actividades de la Vida Diaria y Actividades Instrumentales de la Vida Diaria; educación para el autocontrol de enfermedades para personas con Parkinson y sus cuidadores; enfoque de grupo supervisado; y manejo de factores personales.

**Conclusión:**

la atención de enfermería a las personas con Parkinson en el nivel primario implica esencialmente acciones que incluyen brindar cuidados enfocados a nivel individual y grupal, incluyendo la evaluación clínica, educación del paciente, participación del paciente en el contexto social de la atención y desarrollo de relaciones positivas con los miembros de la familia y los cuidadores.

## INTRODUCTION

The current health scenario, with epidemiological changes, population aging and increased prevalence of chronic non- communicable diseases (CNCDs), requires nurses to seek advanced levels of knowledge and skills on clinical practice in Primary Health Care (PHC), towards the implementation of a comprehensive care approach that combines efforts to manage the physical and psychosocial disabilities of the disease, while promoting the provision of health care and well-being throughout users’ and their families’ lives^(1)^.

Among the CNCDs, neurodegenerative diseases, together with the rapid aging of populations, present themselves as challenges for the health system, since patients require special care and therapies to face the impact of functional limitations on activities of daily living (ADL) and social participation^(2)^. In relation to neurodegenerative diseases, Parkinson’s disease (PD) stands out for its incidence and prevalence, a chronic progressive neurological condition caused by the loss of dopaminergic neurons in *substantia nigra pars compacta* (SNc)^(3)^.

Among health professionals, nurses have been recognized as key professionals in patients’ first contact with health care and, in the care of people with PD. They are capable of collaborating, together with other health care team members, to improve quality of life through a standardized care plan that may include mobility, elimination, and rest/sleep needs, providing care in health promotion and prevention of complications related to the neurodegenerative disease process^(4,5)^.

However, despite the variety of studies on the subject in the hospital area, specialized neurorehabilitation centers and specific scenarios for the role of expert nurses in PD, there has been little discussion about nursing practice for people affected with the disease in PHC^(5)^. The interventions most described in the literature by PD nursing specialists in the context of PHC involve self-management skills that focus mainly on education, emotional support^(4)^ and palliative care^(5)^. The relevance of this study lies in the possibility of expanding knowledge about PHC nurses’ practices and understanding the attributions and implications of this professional in continuity of care for people with PD in this health context.

For this reason, a scoping review was carried out, aiming at mapping and analyzing the scientific literature on nursing care aimed at people with PD in PHC.

## METHOD

This is a scoping review, conducted in accordance with the Joanna Briggs Institute (JBI)^(6)^ methodological guidelines for scoping review and reported in accordance with the Preferred Reporting Items for Systematic reviews and Meta-Analyses extension for Scoping Reviews (PRISMA-SC). The approach did not deviate from the previously published scoping review protocol^(7)^.

### Research Question Identification

The review question was formulated according to the PCC mnemonic. The question elements were as follows: P – Population = people with PD; C – Concept = nursing care for the person with PD; C – Context = PHC. The research question was, therefore: what is the nursing care provided to people with PD in the PHC context?

### Information Source and Inclusion Criteria

This scoping review considered primary quantitative, qualitative and mixed methods studies and all types of secondary studies, such as systematic, scoping, integrative, narrative reviews, among others. Moreover, this review considered studies exploring care provided by nurses alone or in a multidisciplinary approach. Articles in English, Spanish and Portuguese were included, and there was no restriction regarding the diagnostic criteria for PD. No publication date restrictions were made, as the review aimed to report all relevant literature.

Publications that did not detail nursing attributions or care to the person with PD, or those in which nursing care took place in recruitment contexts, such as a rehabilitation center, hospital or nursing home/long-stay institution or similar, and studies with a focus in nursing education were not included.

### Search Strategy

Following JBI recommendations, three steps were used for search strategy. First, limited prior research in PROSPERO, Cochrane Database of Systematic Reviews, JBI Evidence Synthesis, Public Medline (PubMed) and Cumulative Index to Nursing & Allied Health Literature (CINAHL) was performed to identify articles on the topic and analyze text words contained in the titles and abstracts. The index terms used to describe the articles were used to develop a complete search strategy. No systematic or scoping reviews addressing the review question were identified.

Second, a search was performed using the keywords and index terms identified in the databases included: PubMed, CINAHL, Web of Science (WoS), SciVerse Scopus (Scopus), Nursing Database (BDENF), Literature Latin American and Caribbean in Health Sciences (LILACS) and Scientific Electronic Library Online (SciELO), between July and October 2020, updated November 2021. Third, the reference lists of all identified documents were searched for relevant studies, however, no additional references were found for inclusion.

In order to maximize the search accuracy, the searches were designed in collaboration with an expert health science librarian, designing medical subject headings from MEDLINE (MeSH), Descriptors in Health Sciences (DeCS), and terms and keywords were adapted to each database searched according to such descriptors.

**Chart 1.**
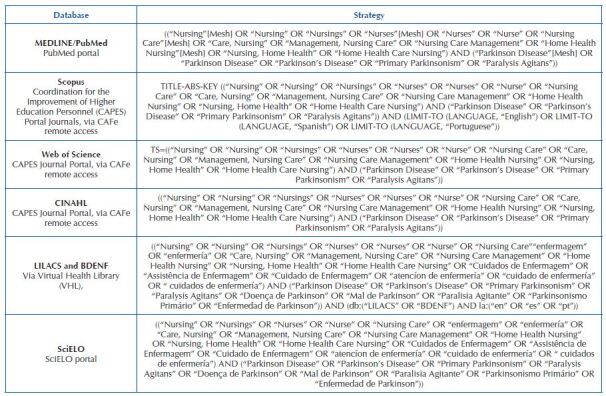
Database search strategy. Florianópolis, SC, Brazil, 2020.

It is noteworthy that the descriptor “Primary Health Care” and its related terms were used in a first search strategy; however, due to the small number of articles found, it was decided, together with the librarian, to remove such terms, analyzing the PHC context in the titles and abstracts as one of the inclusion criteria for article selection. The search strategy for each database is listed in Chart 1.

### Study Selection

After search, two independent reviewers imported the identified records to Mendeley Web Importer for reference management and duplicate removal. Titles and abstracts were then screened for assessment according to inclusion criteria. The selected studies’ full text was retrieved and assessed in detail using the same criteria.

### Data Extraction

The data from included articles were extracted using a data extraction tool developed for this scoping review and published previously in the protocol. The data extracted included specific information on: title, year of publication, country of origin, design, number of participants in the study, area of nursing care addressed and nursing activities performed.

Two trained reviewers independently extracted data from the publications. Any divergences that arose between the two reviewers were resolved through discussion or with a third reviewer.

### Data Synthesis

Following JBI recommendations for analysis, the extracted data were grouped to reflect the main or recurrent themes related to the objective of the review, which, in this case, were the care provided by nurses in the context of PHC. We analyzed studies included in each area of fundamental care to identify which nursing care was most frequently explored^(8)^. The results are presented in charts and figure in descriptive format, followed by narrative synthesis.

## RESULTS

**Figure 1.**
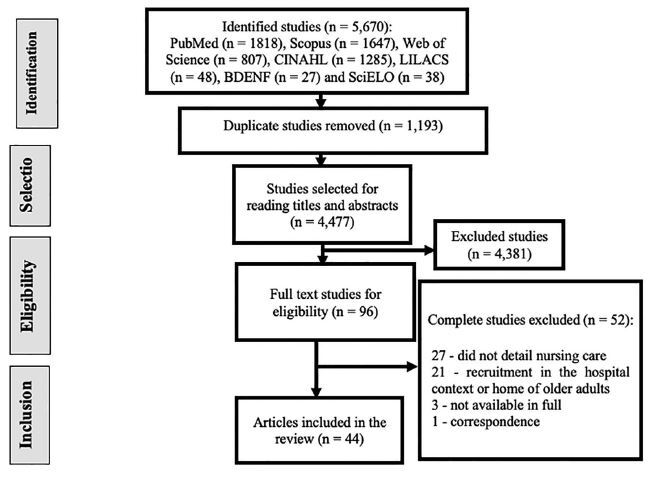
Flow diagram of the study selection process for the scoping review adapted from the Preferred Reporting Items for Systematic Review and Meta-Analyses (PRISMA). Florianópolis, SC, Brazil, 2021.

The research initially resulted in 5,670 publications, of which 4,477 remained for the selection process for titles and abstracts after duplicate removal. At this stage, 4,381 were excluded because they did not meet the pre-established criteria, resulting in the selection of 96 studies for full-text assessment. At this stage, 52 studies were excluded for the reasons listed in Figure 1. Finally, 44 articles that met the eligibility criteria were included in this review. Figure 1 presents the detailed selection process.

### Characteristics of Studies Included

The review consisted of 44 publications published between 1971 and 2021. A total of 43 studies were unicentric, most of them from the United States of America (n = 17), followed by the United Kingdom (n = 8), South Korea (n = 4), Brazil (n = 3), and Spain (n = 2). Canada, Italy, Germany, Denmark, Sweden, Netherlands, Turkey, Singapore, Hong Kong had one study each. Only one study was multicenter, with a partnership between the centers of Japan, Thailand, Canada, Denmark, the United Kingdom and the Netherlands.

There is growing interest and research in the area with emphasis on 2019 and 2020. Chronologically, the first three studies analyzed in this review were conducted in the United States. The first was a case report of a patient with PD dating from 1971, then, only after 16 years, another study related to the theme was carried out, being an experimental study.

**Chart 2.**
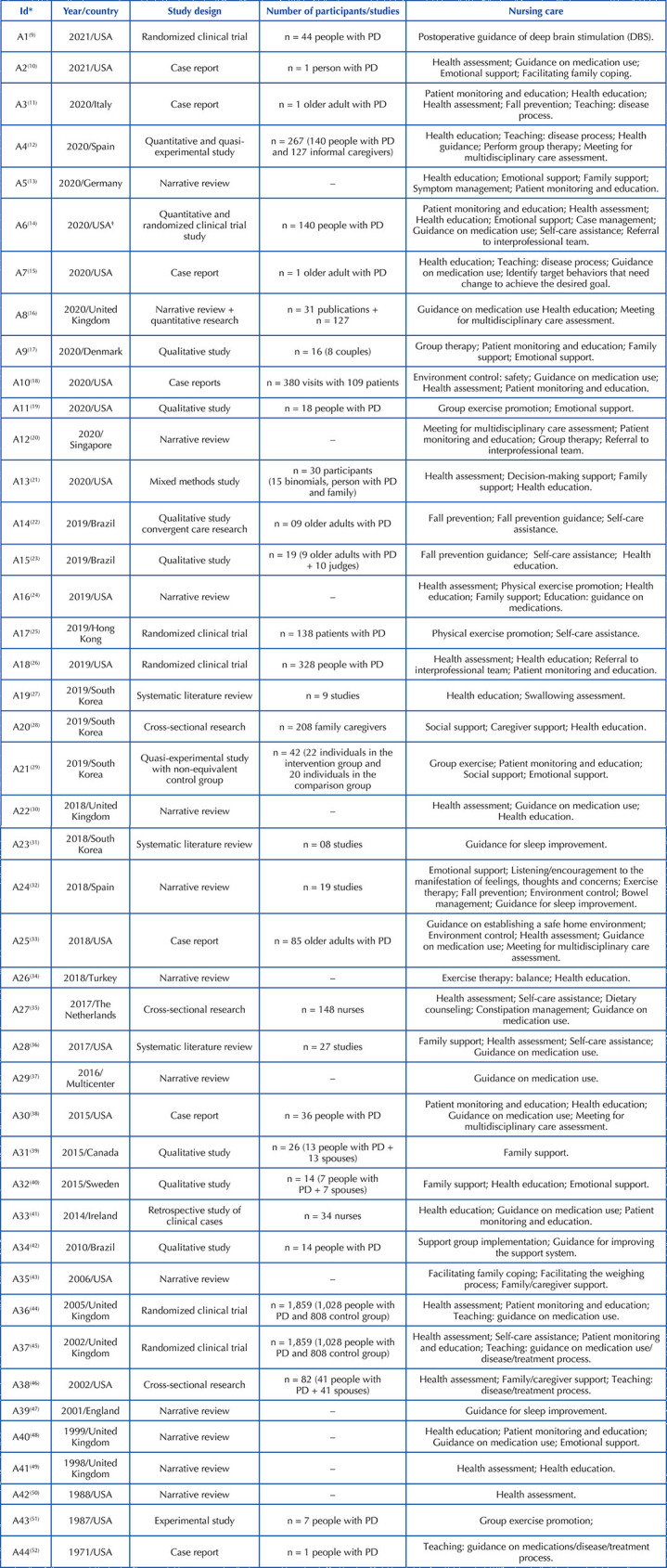
Study characteristics included in the scoping review. Florianópolis, SC, Brazil, 2021.

Regarding the method used, thirteen were quantitative studies, twelve narrative reviews, seven qualitative, seven case reports, three systematic literature reviews, and one study of mixed methods. The included studies targeted people diagnosed with PD and/or their relatives/spouses/caregivers; however, few considered the clinical stage of PD when detailing the sample recruitment. Details can be found in Chart 2.

*Id – identification, Article A followed by sequential number; ^†^USA – United States of America.

### Nursing Care

**Figure 2.**
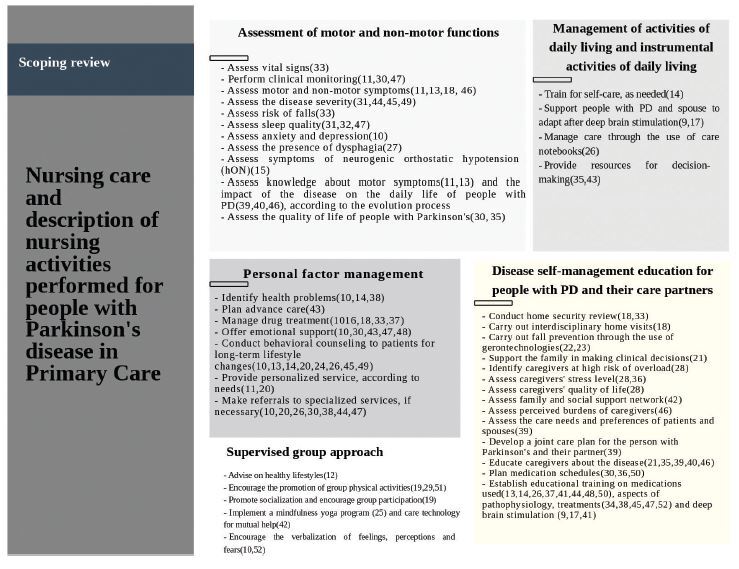
Nursing care and description of nursing activities performed by people with Parkinson’s disease in Primary Care. Florianópolis, SC, Brazil, 2021.

Fifteen studies included in this scoping analysis were reviews, consequently, most of actions performed comprised more than one area of nursing care. The results indicated that nurses focus on improving people with PD’s well-being, and their care partners through health assessment, functional disability management and health education. The nursing care and description of nursing activities performed to people with PD in primary care can be found in Figure 2.

### Assessment of Motor Functions and Non-Motor Functions

Studies^(11,13,15,18,27,30,31,32,33,35,39,40,44–47,49)^ on assessment of motor and non-motor functions revealed that assessment skills of clinical condition, tracking of motor and non-motor symptoms in the various stages of the disease, and global assessment, through specific scales, such as the Movement Disorder Society Unified Parkinson’s Disease Rating Scale, Part III Motor Examination (MDS-UPDRS-III), Parkinson’s Disease Questionnaire–8 (PDQ-8), Parkinson’s Disease Questionnaire–39 (PDQ-39), as well as the use of physical and cognitive domain assessment tests, such as Timed Up and Go test (TUG), Montreal Cognitive Assessment (MoCA) and depression scales are required.

### Management of Activities of Daily Living and Instrumental Activities of Daily Living

The results^(14,17,26,35,43)^ showed self-care support as an important strategy to improve the autonomy and well-being of people with PD, and the most explored themes focused on improving health education to self-care for food, bathing, intimate hygiene and dress. Through clinical assessment and training, nurses will be able to monitor ADL and instrumental activities of daily living (IADL) of people with PD, to assist them in promoting independence and performance of household tasks.

Another common point, cited in studies^(17,24)^, but, to a lesser extent, was the nurses’ ability to instruct people with PD to cope with stress and other psychological demands, as dyskinesias and motor complications can impact the activities or social interactions of people who experience the debilitating effects of the disease.

### Education for Self-Management of the Disease for People with Parkinson’s Disease and Their Care Partners

Clinical evidence^(13,14,17,18,21,22,23,24,28,30,33,34,35,36,37,38,39,40,41,42,44–46,48,50,52)^ available on self-management education of the disease for people with PD and their care partners involves promoting health for family members and caregivers, providing family education^(13,14,24,37,41,44,48,50)^ and safe sharing space for conversations on sensitive issues ranging from sexual issues of the couple after diagnosis to issues related to palliative care for patients at the end of life^(39)^.

Findings^(21,28,36,46)^ indicate that, for promoting education for self-management of the disease, it is necessary to know family functionality, access to community resources, social support network and tracking the participation of each individual involved, being part of nursing care and directly related to people with PD’s quality of life.

Another relevant aspect in self-management education aimed at people with PD and their care partners is home visits. The reviewed studies indicated that, in the interdisciplinary team, nurses lead home care, and are responsible for managing the promotion of a safe home environment. The activities imply monitoring changes in symptoms, problems with medication, identifying people at high risk of falls and issues related to social support and people with PD’ and their families’ health.

### Group Supervised Approach

Group supervised activity was shown to be collaborative in order to achieve positive changes in the health outcomes of people with PD. The nursing activities described in the studies were related to physical-functional training^(19,29,51)^, promotion of socialization^(12,25,42,52)^, support group meetings and mutual help^(42)^, and yoga mindfulness program for anxiety reduction^(25)^.

However, the seven articles^(12,19,25,29,42,51,52)^ included in the theme underestimated collective care in PD, because the actions described in the studies do not go into depth in characterizing the health situation of the territory, compromising the joint construction of the diagnosis of the area and the identification of the real health promotion activities, intersectoral actions, of participation and social control for people living with the disease, important aspects in carrying out supervised group activities.

### Personal Factor Management

In this review, personal factor management^(11,13,14,16,18,20,24,26,30,31,37,38,43–45,47–49)^ was perceived as a fundamental aspect of nursing care to provide significant results to motor and neuropsychiatric symptoms, improving the scores of people with PD’s quality of life. The care consisted of: knowing and assessing clinical and social conditions, providing information on daily routines, treatment management according to predefined care goals^(16,18,33,37)^, focusing on behavioral counseling^(13,14,20,24,26,30,45,49)^ and identification of health problems^(14,38)^.

## DISCUSSION

PD is a neurodegenerative disorder that includes a wide range of motor and non-motor symptoms that can lead to an impact on the well-being of individuals living with the disease^(3)^. Therefore, nurses should be aware of the issues involved in disease progression when providing care to people with PD and their families. This scoping review identified 44 studies on nursing care to meet the needs of people with PD under PHC, which aim at: assessment of motor functions and non- motor functions; management of ADL and IADL; education for self-management of the disease for people with PD and their care partners, supervised approach in group; and personal factor management, thus demonstrating the complexity in relation to nursing care.

The results showed that primary care nurses play an essential role in optimizing the functionality and well-being of people with PD by providing supportive care in the assessment of clinical characteristics, both motor and non-motor, associated with PD severity and duration^(11,13,15,18,27,30–33,35,39,40,44–47,49)^.

As PD manifestations differ between patients and individual needs and priorities are very diverse, nurses should take the time to raise such demands and needs with the support of clinical assessment instruments to obtain care directed to functional reach^(5,8)^. In Brazil, nurses and other PHC health professionals are instrumentalized to monitor elder health and individuals with chronic non-communicable diseases’ health through scales for functional and cognitive assessment, available in protocols and clinical guides of regional or material care, such as the Ministry of Health’s Primary Care Notebooks^(2)^. Although these materials are not specific for PD, the instruments contained in them can assist nurses in clinical assessment and monitoring of neurobehavioral and cognitive disorders, very common in individuals with the disease.

Countries such as the United Kingdom and the Netherlands have successful experiences with specialist nurses’ work in PD, and their specific documents for nursing care in PD establish independence promotion and management as the competence of nurses who assist this public^(35,53)^. For the UK group, nurses should demonstrate knowledge of people with PD’s physiological, social and spiritual needs, assess individuals’ ability to manage their own care and the ability to recognize and describe signs of common problems that affect them and that can compromise self-care, in addition to presenting the concept of anticipatory care, strengthening future decision-making^(53)^.

The clinically heterogeneous and progressive nature of PD^(3)^, requires, in addition to assessment of clinical characteristics, management of ADL and IADL^(14,17,26,35,43)^. It is essential that nurses develop strategies to encourage people with PD in performing ADL and IADL, promoting improvement of quality of life, thus avoiding functional dependence and depressive symptoms^(54)^.

As PD may affect the life not only of the people with the disease, but also for their whole family, education for self- management of the disease for people with PD and their care partners^(13,14,17,18,21–24,28,30,33–42,44–46,48,50,52)^, is an essential part of nursing care. On the theme, it was observed that managing the patient-caregiver binomial includes interventions that aim at the coordination of care for the psychosocial effects of illness that can interfere with relationship interactions, with particular emphasis on strengthening caregiver support networks. In this same direction, authors^(55)^ argue that being diagnosed with neurodegenerative disease is an experience that changes not only individuals’ life with the disease, but also of care partners, making it important to focus on the impacts of PD on family members and caregivers so that they do not become potential patients.

According to revised studies^(11,13,14,17,18,20,26,29,38,41,44,48)^, home care in the telenursing modality proved to be a useful tool to ensure meeting patients’ needs and continuity of care. This resource has been used by PHC nurses, providing users with guidance by phone or videoconference, action planning, home visits, multidisciplinary articulation and referral to specialized services^(4,56)^. In the current scenario, in times of social distancing imposed by COVID-19, teleservice can be a useful tool to maintain the care of people with PD.

A fundamental pillar of PHC, home visit provides proximity of health team professionals to those who cannot reach the service due to the personal or geographical limitations of the territory^(54)^. As with frail older adults, due to physical limitations, people with intermediate and advanced PD benefit from regular visits from professionals assessing the possible risks and limitations of their home environment and access to the territory.

In this review, seven studies^(12,19,25,29,42,51,52)^ reported the group supervised approach. In this subject, a Brazilian^(54)^ study on advances and challenges of health care of older adults with chronic diseases in PHC identified that the formation of groups is an essential strategy in the treatment of chronic diseases, as it allows reflection on the illness process and the factors involved, enabling the fostering of forms of self-care and lifestyle changes.

For the authors^(54)^, when care occurs in an integrated way with the Expanded Family Health and Primary Care Center (NASF-AB - *Núcleo Ampliado de Saúde da Família e Atenção Básica*) professionals, it is possible to achieve significant health results of participants, allowing the reduction of the number of referrals for secondary care. Therefore, nurses should benefit from these interactive spaces to promote socialization and healthy lifestyles through support for group participation, encouragement of verbalization and behavioral counseling for changes in long-term lifestyle.

Considering the studies^(11,13,14,16,18,20,24,26,30,31,37,38,43–45,47–49)^ that examined personal factor management, understanding and identifying the health problem of people with PD from the perspective of nursing will ensure individualized actions aimed at allowing them to maintain or regain control of important aspects of their lives. This, in turn, will help involve them in clinical decisions and in setting therapeutic goals during consultations, improving health outcomes and, consequently, their quality of life and of their caregivers.

Our findings encourage a collaborative approach to primary care that prioritizes multisectoral actions for health promotion and education at the individual level, focusing on the specificities of each patient and collective actions such as holding groups aimed at people with PD and their families, strengthening the ability to prevent complications and self-care^(54,56)^.

The multifaceted nature of PD demands interventions from nurses aimed at educating and supporting patients in maintaining a safe and active lifestyle through the prevention of functional decline and referral to professionals from the interdisciplinary team, especially with a focus on physical exercise, speech therapy, and occupational therapy to decelerate motor and neural degeneration^(20,26,44,47)^. In this regard, the support of NASF professionals in the Brazilian PHC scenario stands out, in which a multidisciplinary approach is carried out, with case discussion and care planning, which is so necessary and important due to the complexity of PD^(54)^.

Although the role of PHC nurses in caring for people with PD was not the focus of this review, it was addressed in studies^(11,14,20,24)^ included in personal factor management that nurses help people with this complex condition to manage their numerous medications, provide information on how to live with the disease, in addition to promoting emotional support to patients and family members. These findings are similar to those found in other reviews^(4,57)^ on clinical integration in PD, noting that, depending on the context of health service, nurses will have different roles and attributions in the care of this patient. However, some assignments are generally common, such as: case management; assessment and guidance on medication adherence; provision of information; Health education; psychosocial support; aid in the development of coping skills; and caregiver support.

Specific clinical competences reinforce PAHO’s proposal of the need to expand the role of nursing in PHC in Latin American countries, emphasizing that new professional profiles, such as advanced practice nurses, are essential to take over the demands of the role with autonomy and offer high quality care to patients^(1)^.

### Study Limitations

As a characteristic of language-limiting reviews, it is possible that some relevant studies have not been contemplated, however, we feel sure that any lost studies do not substantially alter the pattern of the findings. We have not established any time constraints, so outdated documents have also been recovered. Finally, the quality of the 11 narrative reviews included in this study represents a limitation that should be recognized, since, based on the methodological framework used, we did not assess the reports’ methodological qualities.

### Implications for the Advancement of Scientific Knowledge for Health and Nursing

This article points to nursing care that can be performed in the primary scope of health care of people living with PD, assisting nurses in the development of competencies necessary for clinical decision-making. Furthermore, the nursing care listed in this study may be useful to direct individual-level self-management plans throughout the course of the disease, providing clarity on the role of nursing in the various aspects of PD care in the PHC context.

Although this review reveals the growth of studies on the subject in recent years, only some clinical trials^(9,25,26,44)^ assessed the effectiveness of care, demonstrating the need for future high-quality research to generate strong evidence base for the implementation of nursing care at different stages of the disease, distinguishing which identified care works for specific subgroups of this population. Furthermore, understanding collective care practices and their complex relationship in models of care for people living with PD in the territory is an important next step in research in the face of the growing prevalence of the disease, with participation and social control as central elements that should be assessed to characterize the health situation of this population.

## CONCLUSION

This scoping review demonstrates nursing care to people with PD in the PHC environment as complex and multidimensional, emphasizing actions that include individual-centered and group-centered care, covering clinical assessment, patient education, involvement of people with PD in the social context of care and development of positive relationships with family members and caregivers.

As the most important care performed by nurses, in the primary health context, was related to the assessment of symptoms at each clinical stage and educational strategies for managing and coping with the disease, additional work is needed to examine the impact and clinical effectiveness of this care on the health and well-being of people with PD, especially in places where they do not have access to specialized neurorehabilitation services, or in contexts where these services are scarce.

## Financial support

Cássia Baldini Soares

## Financial support

*Fundação de Amparo à Pesquisa e Desenvolvimento Científico e Tecnológico do Maranhão –* FAPEMA provided for in Public Notice 046/2017 – Doctorate in the country and abroad – Doctoral Scholarship, Process BD-08736/17
